# Cardiovascular Protective Effect of Metformin and Telmisartan: Reduction of PARP1 Activity via the AMPK-PARP1 Cascade

**DOI:** 10.1371/journal.pone.0151845

**Published:** 2016-03-17

**Authors:** Fenqing Shang, Jiao Zhang, Zhao Li, Jin Zhang, Yanjun Yin, Yaqiong Wang, Traci L. Marin, Brendan Gongol, Han Xiao, You-yi Zhang, Zhen Chen, John Y-J Shyy, Ting Lei

**Affiliations:** 1 Cardiovascular Research Center, School of Basic Medical Sciences, Xi’an Jiaotong University Health Science Center, Xi'an, China; 2 Department of Cardiology, First Affiliated Hospital of Xi’an Jiaotong University, Xi’an, China; 3 Departments of Cardiopulmonary Science and Anatomy, Schools of Allied Health and Medicine, Loma Linda University, Loma Linda, CA, United States of America; 4 Institute of Vascular Medicine, Peking University Third Hospital, Beijing, China; 5 Department of Medicine, School of Medicine, University of California, San Diego, La Jolla CA, United States of America; 6 Department of Pathology, School of Basic Medical Sciences, Xi’an Jiaotong University Health Science Center, Xi'an, China; The Chinese University of Hong Kong, HONG KONG

## Abstract

Hyperglycemia and hypertension impair endothelial function in part through oxidative stress-activated poly (ADP-ribose) polymerase 1 (PARP1). Biguanides and angiotensin II receptor blockers (ARBs) such as metformin and telmisartan have a vascular protective effect. We used cultured vascular endothelial cells (ECs), diabetic and hypertensive rodent models, and AMPKα2-knockout mice to investigate whether metformin and telmisartan have a beneficial effect on the endothelium via AMP-activated protein kinase (AMPK) phosphorylation of PARP1 and thus inhibition of PARP1 activity. The results showed that metformin and telmisartan, but not glipizide and metoprolol, activated AMPK, which phosphorylated PARP1 Ser-177 in cultured ECs and the vascular wall of rodent models. Experiments using phosphorylated/de-phosphorylated PARP1 mutants show that AMPK phosphorylation of PARP1 leads to decreased PARP1 activity and attenuated protein poly(ADP-ribosyl)ation (PARylation), but increased endothelial nitric oxide synthase (eNOS) activity and silent mating type information regulation 2 homolog 1 (SIRT1) expression. Taken together, the data presented here suggest biguanides and ARBs have a beneficial effect on the vasculature by the cascade of AMPK phosphorylation of PARP1 to inhibit PARP1 activity and protein PARylation in ECs, thereby mitigating endothelial dysfunction.

## Introduction

Endothelial function is impaired under pathophysiological conditions such as hyperglycemia and hypertension, in part because of an imbalanced redox state. Induced by oxidative stress, poly (ADP-ribose) polymerase 1 (PARP1) plays an important role in DNA repair and maintenance of genome stability. Although mild activation of PARP1 can be protective and promote cell survival, excessive and sustained oxidative stress can cause overactivation of PARP1, which escalates the oxidative stress and stimulates pro-inflammatory and necrotic responses [1]. At the expense of NAD^+^, PARP1 synthesizes PAR for "PARylation" of itself and other nuclear and cytoplasmic proteins, which depletes cellular NAD^+^ and ATP and activates transcription factors such as NF-κB and AP-1 and inactivates SIRT1 deacetylase [2–5]. In addition to NF-κB activation, PARP1 exerts its pro-inflammatory effect by binding to the B-cell lymphoma 6 (Bcl-6) intron 1 to suppress the expression of Bcl-6 protein [6].

Hyperglycemia, angiotensin II (Ang II), and oxidized low-density lipoprotein activate PARP1 in vascular endothelial cells (ECs), with attendant increase in oxidative and inflammatory stresses [7]. By contrast, inhibition of PARP1 in ECs protects against free radical-induced cell death [8]. *In vivo*, mice with PARP1 ablation are spared from hyperglycemia-induced endothelial dysfunction [9]. Furthermore, genetic ablation of PARP1 or administration of a PARP1 inhibitor (e.g., PJ-34 and TIQ-A) to ApoE^-/-^ mice, led to decreased atherosclerosis [10–12], which suggests an atherogenic role for PARP1.

Biguanides (e.g., metformin) and sulfonylureas (e.g., glipizide) are first-line anti-diabetic drugs. Ample evidence indicates that metformin reduces cardiovascular incidents, possibly by improving vascular functions such as flow-mediated dilation (FMD) [13]. Data from clinical studies suggest that patients taking metformin show improved FMD [14]. However, no evidence suggests that glipizide improves EC functions or decreases vascular resistance. Regarding anti-hypertensive drugs, both telmisartan, an antagonist of Ang II type 1 receptor (AT1R) and metoprolol, a selective β1 receptor blocker, have a significant effect on lowering blood pressure. Telmisartan decreases arterial stiffness and atherosclerosis by improving EC functions [15–17], but metoprolol might not have this beneficial effect [18].

By phosphorylating substrates such as endothelial nitric oxide synthase (eNOS), peroxisome proliferator-activated receptor-γ (PPARγ) coactivator 1 (PGC-1), and sterol regulatory element-binding proteins (SREBPs), AMP-activated protein kinase (AMPK) increases endothelial function via enhanced NO bioavailability and mitochondrial biogenesis and decreased inflammatory and oxidative stresses [19]. Although we have previously shown that PARP1 is a putative substrate of AMPK [20], the translational implication of this phosphorylation event in the broad context of vascular diseases and pharmacology has not been explored. With newly developed anti-phospho-PARP1 antibody, we address these questions with emphasis on improved endothelial functions by hyperglycemia and hypertension treatment.

## Materials and Methods

### Materials

Antibodies against pan-AMPKα, phospho-AMPK Thr-172, eNOS, and phospho-eNOS Ser-1177 were from Cell Signaling Technology (Beverly, MA); anti-AMPKα1 and anti-AMPKα2 antibodies were from Abcam (Cambridge, UK); anti-PARP1 and anti-PAR monoclonal antibodies were from Trevigen (Gaithersburg, MD); anti-β-actin antibody was from Santa Cruz Biotechnology (Santa Cruz, CA). Rabbit polyclonal anti-phospho-PARP1 Ser-177 antibody was produced with the sequence “ELGFRPEY(pS)ASQ” by AbMax Biotechnology (Beijing, China). Metformin, glipizide, telmisartan, metoprolol, atorvastatin, AICAR, Compound C and PJ34 were from Sigma-Aldrich (St. Louis, MO). The PARP assay kit was from Trevigen (Gaithersburg, MD). NO detection kit was from Beyotime Biotechnology (Haimen, China).

### Cell culture, adenovirus, and EC infection and transfection

Human umbilical vein endothelial cells (HUVECs) were isolated from human umbilical cords. With patients’ consensus, the cords were obtained from Department of Obstetrics and Gynecology, the First Affiliated Hospital, Xi’an Jiaotong University. The protocol for the isolation of endothelial cells was reviewed and approved by the Ethic Committee of Xi’an Jiaotong University Health Science Center. HUVECs were cultured in medium M199 supplemented with 20% fetal bovine serum (FBS), 1 ng/mL recombinant human fibroblast growth factor, 90 μg/mL heparin, 20 mM HEPES (pH 7.4) and 100 U/mL penicillin-streptomycin. Bovine aortic endothelial cells (BAECs) were a gift from Department of Physiology, Peking University. BAECs were cultured in Dulbecco’s modified Eagle’s medium (DMEM) supplemented with 10% FBS and antibiotics. Cells were maintained in a humidified 95% air, 5% CO_2_ incubator at 37°C. We used a recombinant adenovirus expressing a constitutively-activated form of AMPKα2, hereafter called Ad-AMPK-CA. The parental adenoviral vector, called Ad-null, was used as an infection control. Confluent ECs were infected with recombinant adenoviruses at the indicated multiplicity of infection (MOI) and incubated for 24 hr before experiments. ECs were transfected with various DNA plasmids with the use of Lipofectamine 2000 RNAi Max (Invitrogen).

### RT-qPCR analysis of mRNA

RNA was isolated from cultured cells or tissues by using TRIzol (Invitrogen). For mRNA quantification, total RNA was reverse-transcribed (RT) by use of the iScript cDNA synthesis kit (Invitrogen), followed by quantitative real-time PCR (qPCR) with SYBR Green (Promega) and a 7500 realtime PCR system (Applied Biosystems). The relative level of mRNA was calculated by the ΔΔ Ct method with GAPDH or other appropriate genes as an internal control.

### Western blot analysis

Total proteins were extracted by use of RIPA buffer (6.5 mM Tris, pH 7.4, 15 mM NaCl, 1 mM EDTA, 0.1% SDS, 0.25% sodium deoxycholate, 1% NP-40). Bicinchoninic Acid reagents (Thermo Scientific) were used to measure the protein concentration. Equal amounts of proteins were separated by SDS-PAGE and transferred to PVDF membranes. The blots were immunoreacted with primary antibodies and secondary antibodies conjugated with horseradish peroxidase. Protein bands were visualized by enhanced chemiluminescence detection and the intensity was quantified by use of Scion Image software.

### PARP1 activity measurement and NO generation detection

For the colorimetric PARP1 activity assay, EC nuclear extracts were incubated with histones and PARP cocktail in a strip well format. The PARP1 activity was then assessed by incorporation with biotinylated poly(ADP-ribose), which was quantified by the reading the absorbance at 450 nm. Serially diluted PARP-HSA (high specific activity) standards were used in parallel to generate the standard curve for subsequent calculation. EC-derived NO was measured in condition media by using Griess reagents following the standard protocol.

### Animal experiments

The animal experiments were approved by the Institutional Animal Care and Use Committee of Xi’an Jiaotong University (No. XJTULAC2014-208). All animals were housed in colony cages (Animal Care Systems, Centennial, Colorado, USA) with a 12-hr light/12-hr dark cycle *ad libitum*. We monitored the mouse and rat body weight, teeth, fur, and behavior on the daily base. According to our close observation, there was no mortality during the experimental period. At the end of experiments, all animals were euthanized by CO_2_ and tissues were collected afterwards for analyses. All treatments were by oral gavage. C57BL6 mice were purchased from Experimental Animal Center of Xi’an Jiaotong University. Eight- to twelve-week-old male C57BL6 mice were treated with metformin (200 mg/kg/day), glipizide (1.3 mg/kg/day), telmisartan (10 mg/kg/day), metoprolol (30 mg/kg/day), or saline as a mock control for up to 24 hr. AMPKα2^-/-^ mice were a gift from Institute of Vascular Medicine, Peking University Third Hospital. AMPKα2^-/-^ mice and their wild-type littermates (AMPKα2^+/+^) in a C57BL6 background were treated with metformin (200 mg/kg/day) for 12 hr. Ten-week-old db/db and age-matched db/m mice were purchased from the Model Animal Research Center of Nanjing University. db/db mice were treated with metformin (200 mg/kg/day) or glipizide (1.3 mg/kg/day) for 2 weeks. Fasting blood glucose was measured by use of the Accu-Check Glucose Meter. Spontaneously hypertensive rats (SHRs) and Wistar-Kyoto (WKY) rats were purchased from Vital River Laboratory Animal Technology (Beijing, China). Ten-week-old male SHRs and WKY rats were fed standard chow diet and treated with telmisartan (10 mg/kg/day) or metoprolol (30 mg/kg/day) for 8 weeks. Systolic arterial pressure was measured by tail-cuff plethysmography.

### Statistical Analysis

Significance of variability was determined by unpaired Student’s *t* test between two groups or ANOVA for multiple comparisons. Data were expressed as mean±SD from at least 3 independent experiments. *p*<0.05 was considered statistically significant.

## Results

### High glucose and Ang II decrease PARP1 Ser-177 phosphorylation in ECs

**Fig 1:**
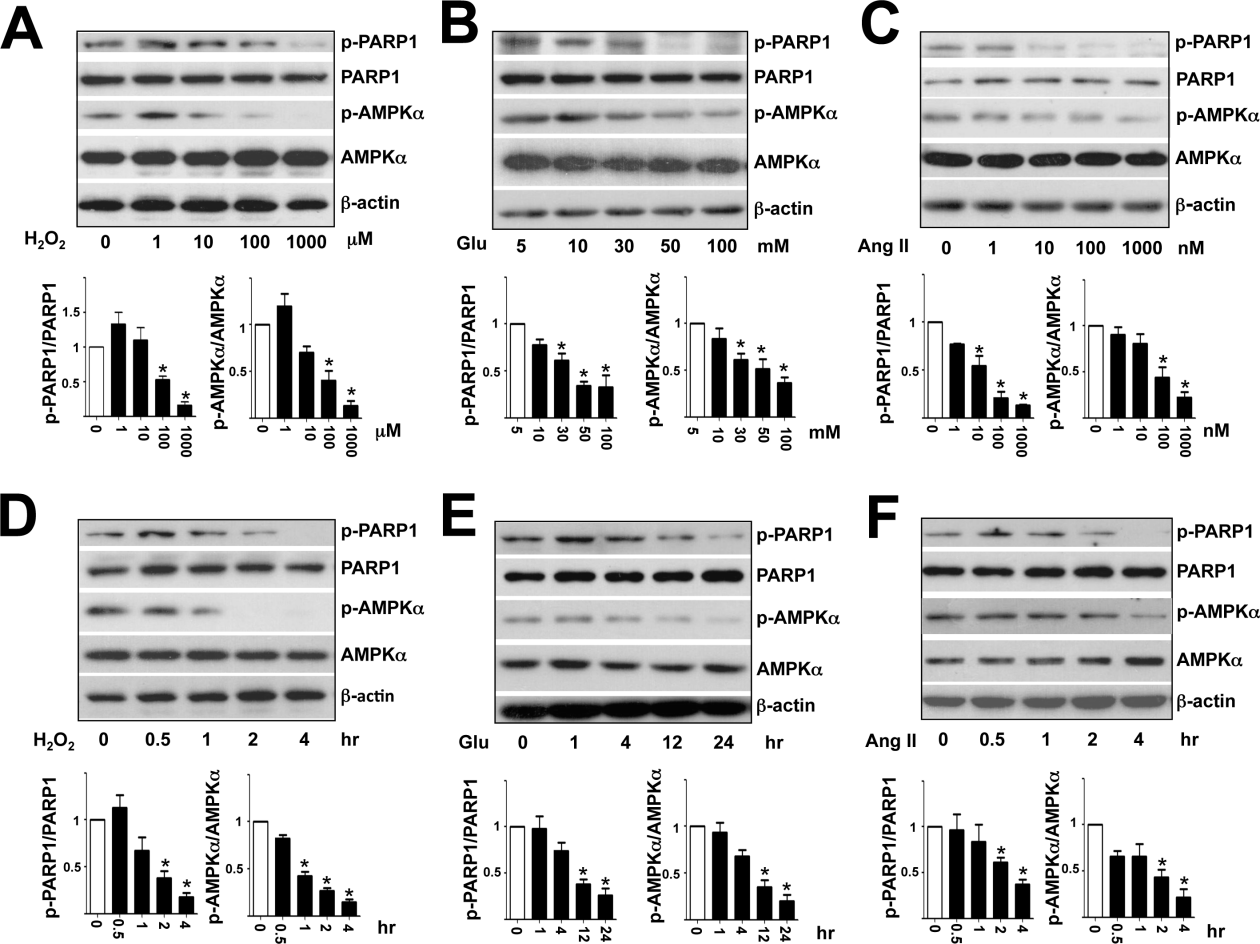
H_2_O_2_, high glucose, and Ang II inhibit phosphorylation of AMPK Thr-172 and PARP1 Ser-177 in HUVECs. Western blot analysis of AMPK Thr-172 and PARP1 Ser-177 in HUVECs treated with concentrations of H_2_O_2_ (A), glucose (B), and Ang II (C) for 4 hr. (D-F) treated with H_2_O_2_ (100 μM), glucose (30 mM), or Ang II (100 nM) for the indicated times. Data are mean±SD ratio of phospho-PARP1 Ser-177 to total PARP1 and phospho-AMPK Thr-172 to total AMPK from 3 independent experiments. *p<0.05 compared to controls.

Given that AMPK activity is inhibited under hyperglycemia and a high level of Ang II, both known to augment oxidative stress in ECs [21,22], we first sought to test whether phosphorylation of PARP1 Ser-177 was also decreased under these conditions with the use of the newly developed anti-phospho-PARP1. As expected, H_2_O_2_, high glucose and Ang II dose- and time-dependently attenuated the phosphorylation of AMPKα Thr-172 and PARP1 Ser-177 in HUVECs (Fig 1). Notably, the use of 30 mM mannitol did not decrease PARP1 and AMPK phosphorylation as that of glucose did (S1 Fig). Further, although H_2_O_2_ at a lower concentration (1 μM) slightly induced AMPK and PARP1 phosphorylation (Fig 1A), these phosphorylation events were decreased at higher concentrations (e.g., 100 and 1000 μM).

### Metformin and telmisartan increase PARP1 Ser-177 phosphorylation

**Fig 2:**
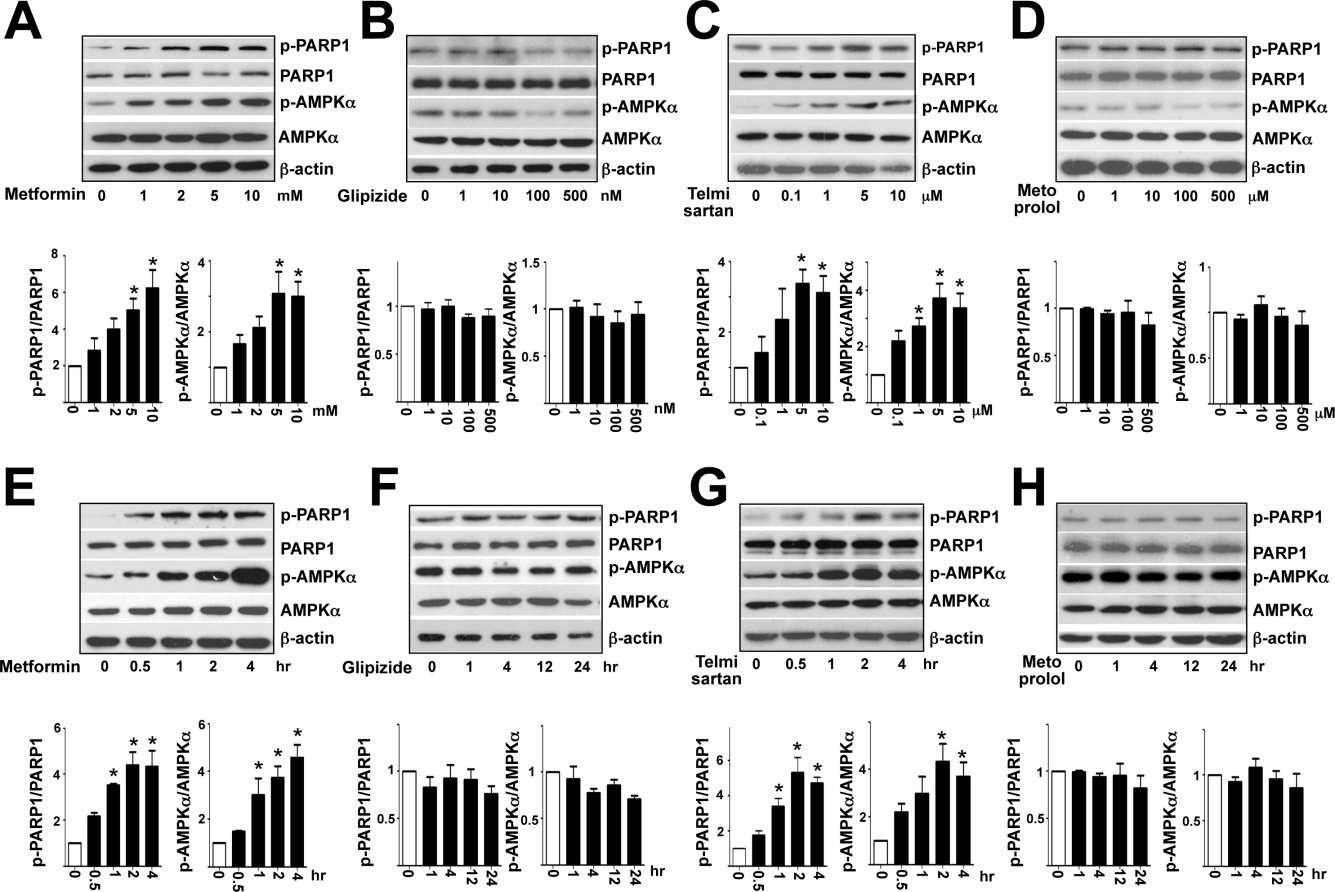
Metformin and telmisartan enhance AMPK and PARP1 phosphorylation in HUVECs. Western blot analysis of AMPK Thr-172 and PARP1 Ser-177 phosphorylation in HUVECs treated with concentrations of metformin (A), glipizide (B), telmisartan (C), and metoprolol (D) for 4 hr or (E-H) metformin (5 mM), glipizide (500 nM), telmisartan (5 μM), and metoprolol (500 μM) for the indicated times. Data are mean±SD ratio of phospho-PARP1 to total PARP1 and phospho-AMPK to total AMPK from 3 independent experiments. *p<0.05 compared to controls.

**Fig 3:**
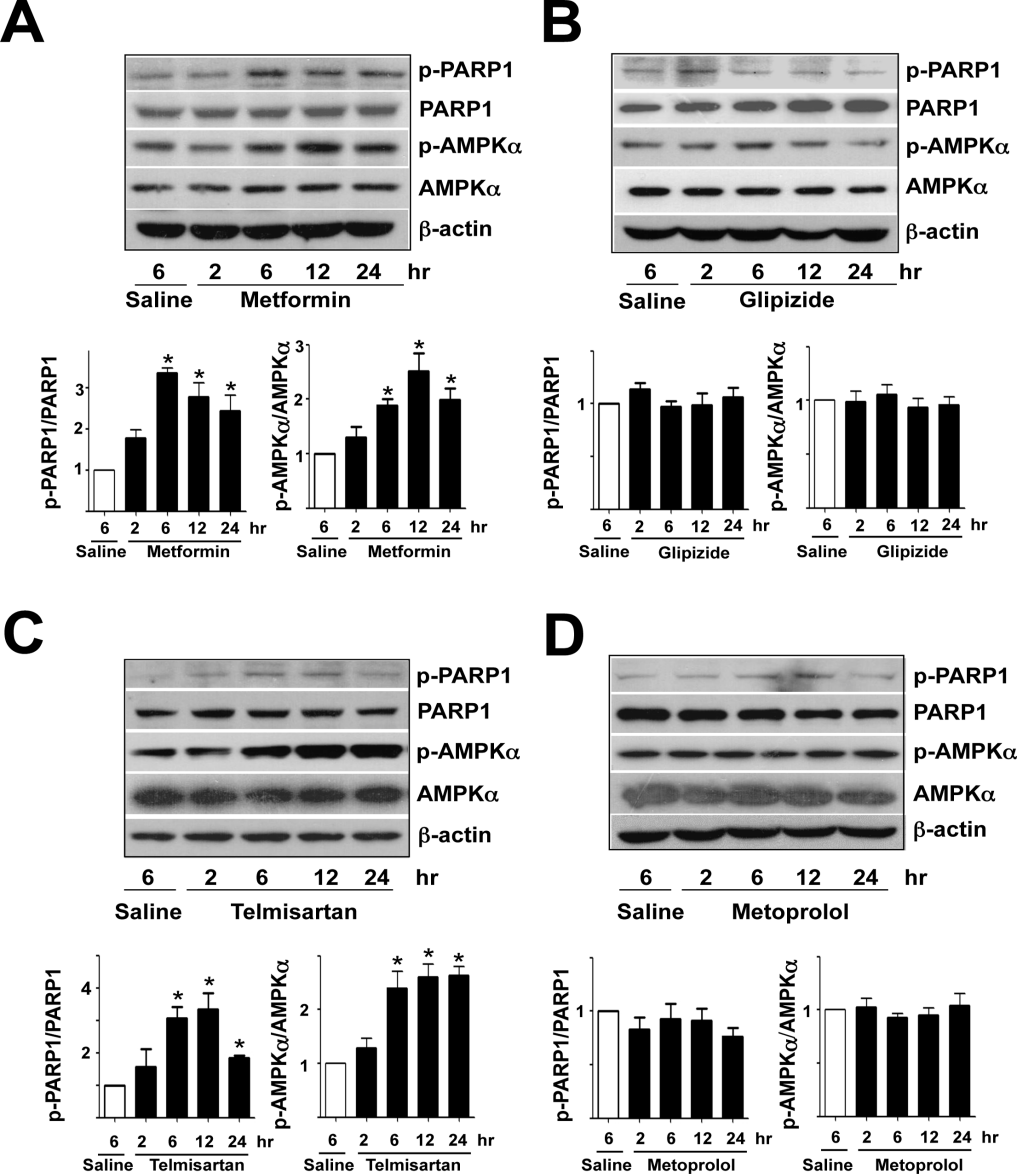
Metformin and telmisartan induce AMPK and PARP1 phosphorylation in mouse aortas. Twelve week-old male C57BL6 mice were administered (A) metformin (200 mg/kg body weight), (B) glipizide (1.3 mg/kg body weight), (C) telmisartan (10 mg/kg body weight), or (D) metoprolol (30 mg/kg body weight) for the indicated times. Control mice received 0.5 ml saline. Western blot analysis of AMPK and PARP1 phosphorylation in aortic extracts from 2 mice pooled. Data are mean ± SD ratio of phospho-PARP1 to total PARP1 and phospho-AMPK to total AMPK (n = 8 mice per group). *p<0.05 compared to controls.

Although metformin and glipizide are first-line drugs to treat type II diabetes, metformin, but not glipizide, has an additional effect on vasculature [23]. Therefore, we examined whether metformin and glipizide could induce PARP1 phosphorylation in HUVECs. Metformin treatment indeed dose- and time-dependently increased the phosphorylation level of PARP1 Ser-177 and AMPK Thr-172 in cultured HUVECs (Fig 2A and 2E). However, cells treated with glipizide showed little increase in PARP1 and AMPK phosphorylation (Fig 2B and 2F). Regarding anti-hypertensive medication, telmisartan improves EC functions, but metoprolol does not seem to benefit the endothelium [17,18]. Similarly, telmisartan, but not metoprolol, induced PARP1 and AMPK phosphorylation (Fig 2C, 2D, 2G and 2H). To recapitulate findings from experiments with cultured HUVECs, we treated C57BL6 mice with metformin, glipizide, telmisartan, and metoprolol. Compared with aortas of control mice receiving saline, aortas of mice with metformin or telmisartan treatment showed increased PARP1 Ser-177 and AMPK Thr-172 phosphorylation (Fig 3A and 3C). However, glipizide and metoprolol administration did not significantly increase the phosphorylation of PARP1 or AMPK (Fig 3B and 3D). Together, results presented in Figs 1–3 suggest that phosphorylation of PARP1 Ser-177 agreed with AMPK activity in ECs under pathophysiological conditions (i.e., elevated oxidative stress) or with pharmacological interventions (i.e., metformin and telmisartan).

### PARP1 Ser-177 Phosphorylation is AMPK-dependent

**Fig 4:**
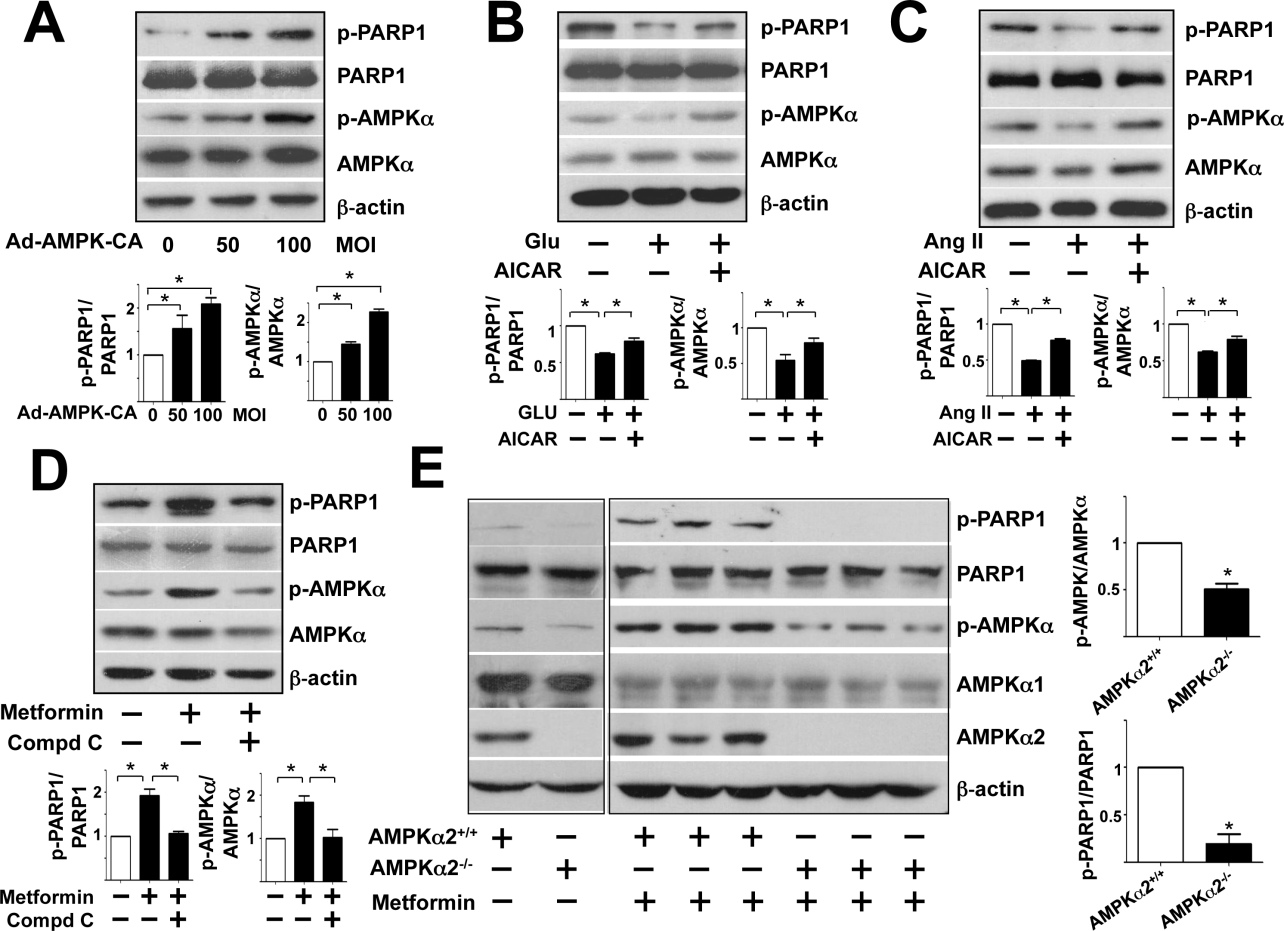
AMPK phosphorylates PARP1 Ser-177 *in vitro* and *in vivo*. Western blot analysis of protein levels in cell lysates and aortic extracts. (A) HUVECs were infected with Ad-AMPK-CA at 50 or 100 multiplicities of infection (MOI) or Ad-null virus at 50 MOI for 24 hr. (B,C) HUVECs were pre-treated with or without AICAR (1 mM) for 30 min before the addition of glucose (30 mM) or Ang II (100 nM) at the indicated concentrations for 4 hr. (D) HUVECs were pre-treated with or without Compound C (15 μM) for 30 min before metformin (5 mM) for 4 hr. (E) AMPKα2^+/+^ and AMPKα2^-/-^ mice were orally administered with or without metformin (200 mg/kg body weight) and aortas were collected after 12 hr. Data are mean±SD ratio of phospho-PARP1 to total PARP1 and phospho-AMPK to total AMPK from at least 3 experiments in A-D and n = 8 animals in E. *p<0.05 compared with controls.

Because PARP1 Ser-177 is a putative AMPK phosphorylation site [20], we investigated whether PARP1 phosphorylation in HUVECs is AMPK-dependent. HUVECs were infected with Ad-AMPK-CA (a constitutively active form for AMPKα2) at different multiplicity of infection (MOI) and control cells were infected with Ad-null at 50 MOI. Ectopic expression of a constitutively active form of AMPKα2 increased the phosphorylation of AMPK Thr-172 and that of PARP1 Ser-177 (Fig 4A). Furthermore, activation of endogenous AMPK by 5-aminoimidazole-4-carboxamide ribonucleotide (AICAR) increased phosphorylation of PARP1 and AMPK in HUVECs under high glucose and Ang II (Fig 4B and 4C). In a complementary experiment (Fig 4D), the increased phosphorylation of AMPK and PARP1 in response to metformin was attenuated by pretreatment with the AMPK inhibitor Compound C.

To explore whether AMPK is involved in the PARP1 phosphorylation *in vivo*, we assessed metformin-induced PARP1 phosphorylation in aortas from AMPKα2^-/-^ mice and their wild-type littermates (i.e., AMPKα2^+/+^). Consistent with results from *in vitro* experiments, the phosphorylation of PARP1 Ser-177 was lower in metformin-administered AMPKα2^-/-^ than AMPKα2^+/+^ mice (Fig 4E).

### PARP1 Ser-177 phosphorylation affects endothelial function

**Fig 5:**
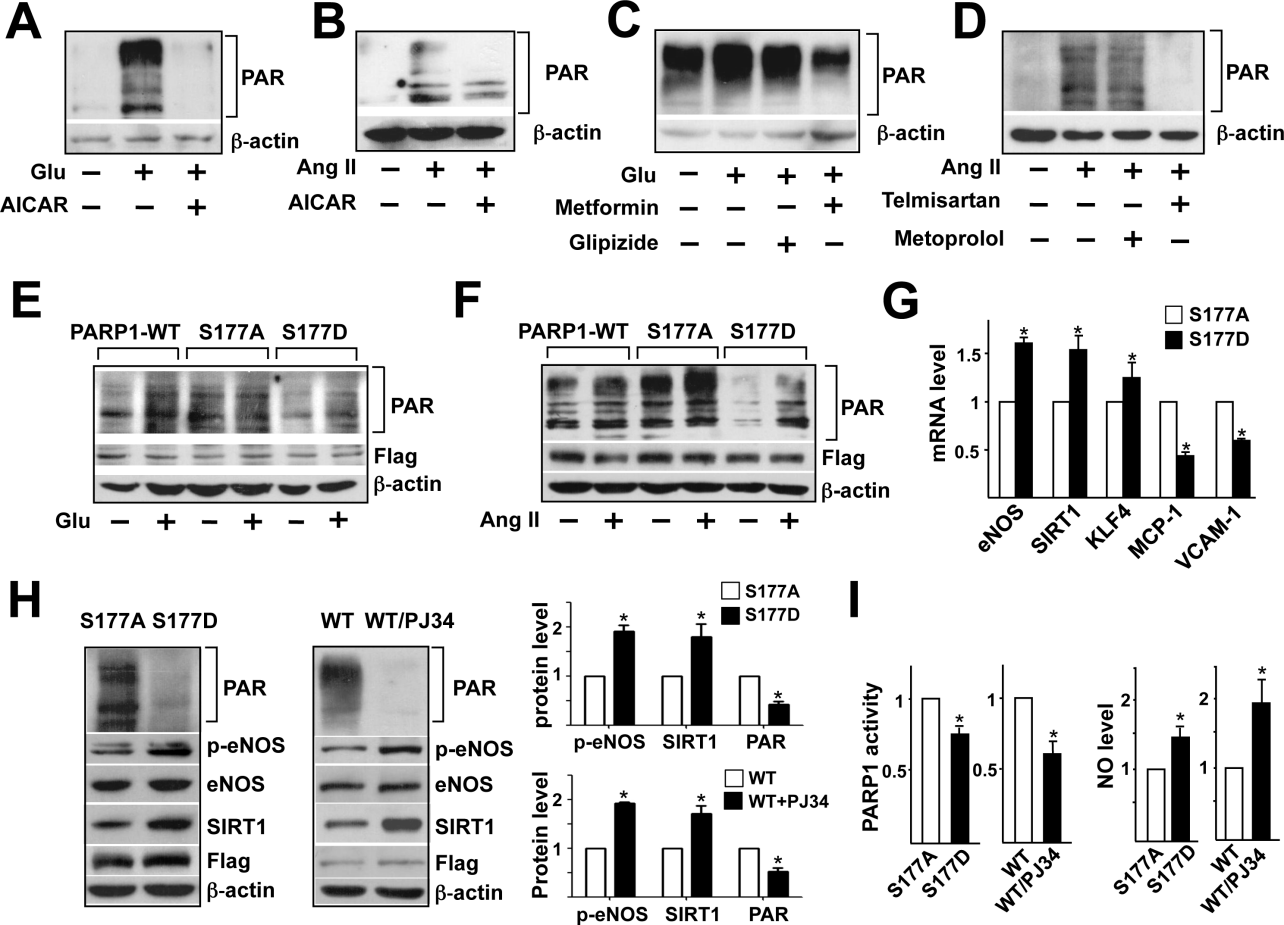
AMPK phosphorylation of PARP1 Ser-177 regulates EC function. (A-F) Western blot analysis of protein PAR in EC lysates. (A, B) HUVECs were pre-treated with or without AICAR for 4 hr before the addition of glucose (30 mM) (A) or Ang II (100 nM) (B) and incubated for another 24 hr. (C) HUVECs were treated with or without metformin or glipizide for 6 hr, then incubated with or without 30 mM glucose for 24 hr. (D) HUVECs were treated with or without telmisartan or metoprolol for 6 hr, then incubated with or without 100 nM Ang II for 24 hr. (E, F) BAECs were transfected with flag-tagged wild-type (WT), S177A, or S177D PARP1 plasmids for 24 hr, then incubated with 30 mM glucose (E) or 100 nM Ang II (F) for 6 hr. (G) BAECs were transfected with S177A or S177D PARP1 plasmid. RT-PCR analysis of mRNA level of eNOS, SIRT1, KLF4, MCP-1, and VCAM-1. (H, I) BAECs were transfected with S177A or S177D PARP1 or WT PARP1 treated with or without PJ-34 (3 μM for 6 hr). (H) Western blot analysis and quantification of protein levels. (I) Measurement of PARP1 activity in nuclear extracts of BAECs and NO level in the cultured medium. Data are mean±SD from at least 3 experiments. *p<0.05 compared with controls.

Next, we examined the role of AMPK phosphorylation of PARP1 Ser-177 in modulating PARP1 activity and related EC function. Because both high glucose and Ang II can activate PARP1 and cause endothelial dysfunction [24,25], we cultured HUVECs under high glucose or Ang II with or without AICAR to examine whether activation of AMPK can inhibit PARP1 activation and consequent PARylation. With 30 mM glucose or Ang II treatment, the protein level of PAR was increased in HUVECs as compared with respective controls (Fig 5A and 5B). Co-incubation with AICAR significantly reduced the high glucose- or Ang II-induced protein PARylation (Fig 5A and 5B). We then compared the effect of metformin versus glipizide and telmisartan versus metoprolol on protein PARylation. Metformin reduced PARylation, and glipizide had little effect on HUVECs under 30 mM glucose (Fig 5C). Similarly, telmisartan, but not metoprolol, decreased Ang II-increased PARylation (Fig 5D). These results are consistent with PARP1 activity assay (S2 Fig).

To confirm further whether AMPK phosphorylation of PARP1 Ser-177 inhibited PARP1 activity and PARylation, we transfected BAECs with plasmids encoding the wild-type, de-phospho-mimetic S177A PARP1, or phospho-mimetic S177D PARP1. High glucose or Ang II increased protein PARylation in BAECs transfected with wild-type PARP1, and overexpression of PARP1 S177A increased PARylation both at the basal level and with glucose or Ang II stimulation (Fig 5E and 5F). In contrast, protein PARylation was lower with PARP1 S177D than S177A or wild-type transfection at the basal level and under glucose or Ang II stimulation. Given the strong inhibition of protein PARylation by PARP1 S177D, we further examined its effect on the expression of genes critical to BAEC function. As expected, the mRNA level of eNOS, SIRT1 and Krüppel-like factor 4 (KLF4) was higher and that of monocyte chemoattractant protein-1 (MCP-1) and vascular cell adhesion molecule-1 (VCAM-1) was lower in BAECs with PARP1 S177D than S177A overexpression (Fig 5G). We also compared p-eNOS Ser-1177 and SIRT1 protein level in BAECs transfected with PARP1 S177A or S177D mutants. In a parallel experiment, wild-type PARP1-transfected BAECs were treated with PJ-34, a PARP1 pharmacological inhibitor. The effect of PARP1 S177D overexpression was similar with PJ-34 treatment, both of which decreased PARP1 activity and protein PARylation but increased eNOS Ser-1177 phosphorylation, SIRT1 expression level, and NO bioavailability (Fig 5H and 5I). Thus, AMPK phosphorylation of PARP1 Ser-177 inhibits PARP1 activity and hence improves endothelial function.

### Metformin and telmisartan activate the AMPK-PARP1 cascade in diabetic and hypertensive animal models

**Fig 6:**
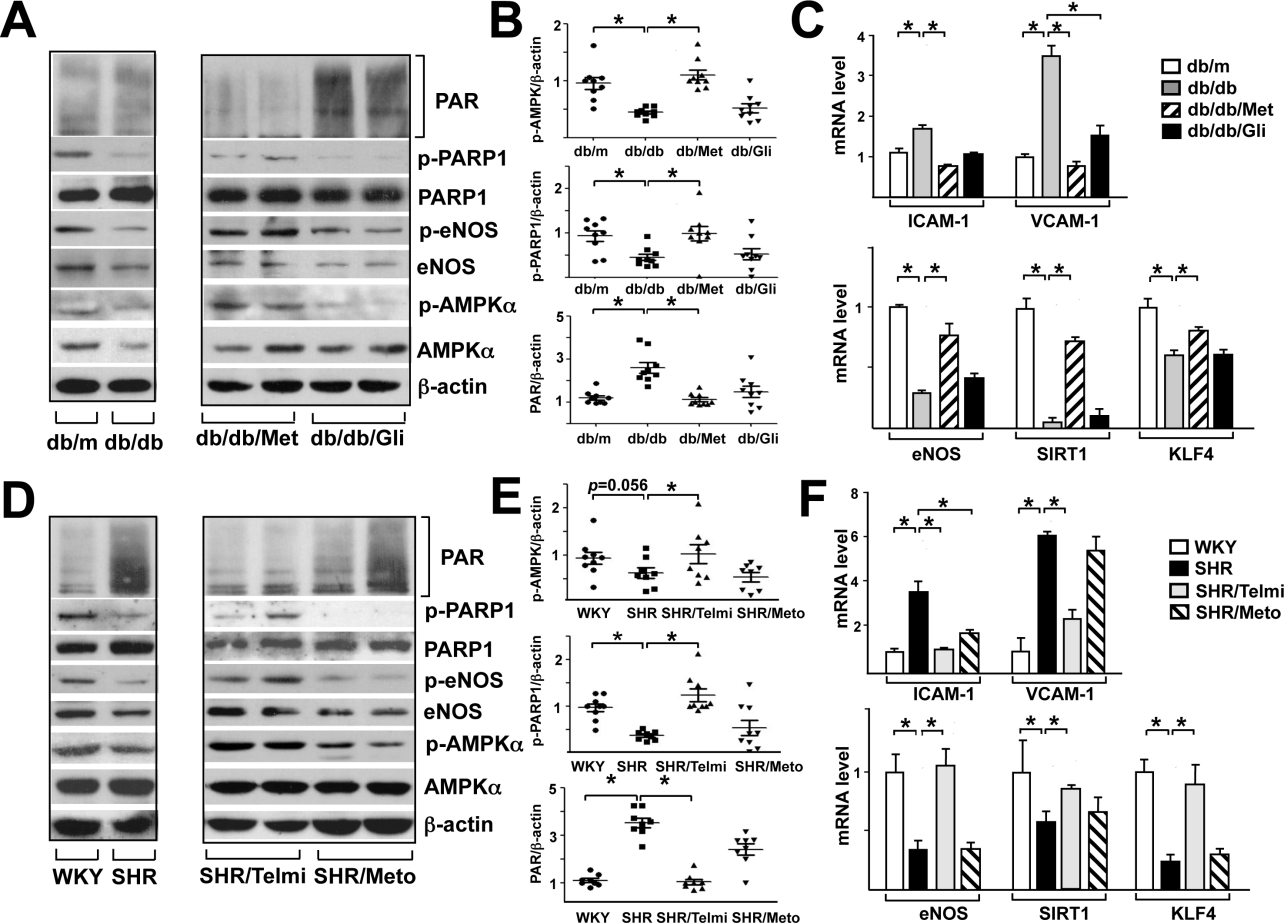
Metformin and telmisartan activate the AMPK-PARP1 cascade in aortic vessel wall of rodents under hyperglycemia and hypertension. db/m and db/db mice were treated with metformin (200 mg/kg/day) or glipizide (1.3 mg/kg/day) for 2 weeks. SHR and WKY rats were treated with telmisartan (10 mg/kg/day) or metoprolol (30 mg/kg/day) for 8 weeks. (A, D) Western blot analysis of protein levels in aortic extracts from various animal groups were analyzed by western blotting with various antibodies as indicated. (B, E) Scatter plots of ratio of phospho-PARP1 to total PARP1, phospho-AMPKα to total AMPKα, and PAR to β-actin for each aortic specimen. (C, F) RT-PCR analysis of mRNA level of eNOS, SIRT1, KLF4, ICAM-1, and VCAM-1 in rodent aortas. *p<0.05 compared with controls.

To provide *in vivo* evidence for the AMPK-PARP1 cascade and its downstream function, we used diabetic and hypertensive rodent models and compared the drug effect of metformin versus glipizide and that of telmisartan versus metoprolol in the long-term (i.e., 2 and 8 weeks). At the basal level, namely untreated groups, db/db mouse and SHR aortas showed lower phosphorylation of AMPKα, PARP1, and eNOS and higher protein PARylation than db/m mouse and WKY rat aortas, respectively (left panels, Fig 6A, 6B, 6D and 6E). Consistent with previous reports, metformin and glipizide had similar glucose-lowering effect in db/db mice (S3 Fig). As well, telmisartan and metoprolol decreased blood pressure in SHR to the comparable level (S4 Fig). However, metformin but not glipizide, increased the phosphorylation of AMPKα, PARP1, and eNOS and decreased protein PARylation in db/db mouse aortas (right panel, Fig 6A and 6B). Telmisartan, but not metoprolol, increased the phosphorylation of AMPKα, PARP1, and eNOS and decreased protein PARylation in SHR aortas (right panel, Fig 6D and 6E). To associate the activation of AMPK-PARP1 with vascular function in various models, we assessed the expression of genes affecting EC function positively (e.g., eNOS, SIRT1, KLF4) or negatively (e.g., ICAM-1, VCAM-1). The mRNA levels of eNOS, SIRT1 and KLF4 were lower and those of ICAM-1 and VCAM-1 higher in db/db mouse and SHR aortas than db/m mouse and WKY rat controls, respectively. Metformin but not glipizide improved EC functions in diabetic db/db mouse aortas, as indicated by decreased expression of ICAM-1 and VCAM-1 and increased expression of eNOS, SIRT1, and KLF4 (Fig 6C). A similar vascular beneficial effect was found in aortas of SHRs receiving telmisartan but not metoprolol (Fig 6F).

## Discussion

Herein, we demonstrated for the first time that AMPK phosphorylation of PARP1 Ser-177 *in vivo*, which contributes to the endothelial protection in the context of anti-hypertensive and anti-diabetic drugs. Although vasculature is directly benefited from the blood pressure and glucose lowering effect, additional therapeutic efficacy of some, but not all, of these drugs may build on the endothelial protection because of this AMPK-dependent PARP1 inhibition. Thus, the major translational implication of this finding is that the vascular protection of metformin and telmisartan occurs in part through activation of the AMPK-PARP1 cascade.

Clinical studies such as the UK Prospective Diabetes Study (UKPDS) have underscored the vasoprotective effect of metformin. In contrast, patients receiving sulfonylurea (e.g., glipizide) did not show a similar extent of cardiovascular protection despite glycemic control [23,26,27]. Vasoprotection with metformin is mediated in part, if not predominantly, by improved EC function [28]. At the molecular level, metformin increased AMPK activity in adipose tissue from diabetic patients, which was not observed with sulfonylurea treatment [29]. Additionally, metformin improves endothelial function in obese mice by inhibition of ER stress through AMPK/PPARδ pathway [30]. Hyperglycemia associated with diabetes mellitus is known to impair endothelial function, as manifested by PARP1 activation and resulting oxidative and inflammatory stresses [24]. Given our findings that metformin can induce AMPK phosphorylation of PARP1 Ser-177 and in turn inhibit PARP1 activation, the AMPK-PARP1 axis may be an important mechanism underlying the vasoprotective effect of metformin. Phenformin, another biguanide class of drug, also activates the AMPK-PARP1 axis in HUVECs (S5 Fig). Metformin is also used to treat polycystic ovary syndrome that is often associated with endothelial dysfunction [31,32]. AMPK phosphorylation of PARP1 Ser-177 may also contribute to the improved EC function in PCOS patients receiving metformin [33].

Although endothelial dysfunction is prevalent in hypertensive patients, the protective effect of anti-hypertensive drugs on the endothelium is less clear than that of biguanides. In the 2014 evidence-based guideline for management of hypertension in adults, the Eighth Joint National Committee (JNC 8) recommended ARBs as a first-line treatment, with β-blockers as later-line alternatives in part because of the higher rate of cardiovascular events associated with β-blockers as compared with ARBs [34]. Several small-scale clinical studies indicated that telmisartan improved FMD in patients with essential hypertension or metabolic syndrome [15,35,36]. As well, the ARBs losartan and irbesartan were found superior to atenolol (a selective β1 blocker) in improving the structure and EC function of resistant arteries [37,38]. Likewise, telmisartan, but not metoprolol, restored eNOS activity and ameliorated oxidative stress in diabetic rats [39,40]. Others showed that telmisartan activated PPARγ in macrophages and ECs [17,41,42], and we demonstrated that telmisartan, but not metoprolol, activated the AMPK-PARP1 axis, which provides additional evidence for the cardiovascular protective effect of telmisartan. This finding is consistent with a report by Sharma et al showing that metoprolol did not change AMPK activity or phosphorylation of acetyl-CoA carboxylase, a canonical substrate of AMPK [43].

Mechanistically, AMPK phosphorylation of PARP1 Ser-177 resembled the pharmacological inhibition of PARP1 by PJ-34 (Fig 5), which reduced PARP1 activity. Given that PARP1 positively regulates NF-κB, p38, and c-Jun N-terminal kinase (JNK) [3,44], AMPK phosphorylation of PARP1 would suppress these pro-oxidative and pro-inflammatory pathways. We found the expression of eNOS, SIRT1, and KLF4 increased and that of MCP-1 and VCAM-1 decreased (Fig 5G), which indicates improved EC function.

Besides AMPK, several kinases have been shown to regulate PARP1. However, these phosphorylation events increase, rather than decrease, PARP1 activity. Extracellular signal-regulated kinase (ERK) can bind to PARP1 and phosphorylate Ser-372 and Thr-373 to enhance PARP1 activity [45,46]. In the context of EC biology, inhibition of ERK can decrease disturbed flow-induced PARP1 activity [47]. As well, JNK can interact with and phosphorylate PARP1 to increase PARP1 activity [48]. With the robust induction of PARP1 by oxidative stress, ERK and/or JNK activation under hyperglycemia and hypertension may contribute to increased PARP1 activity. In contrast, metformin and telmisartan decreased PARP1 activity via the AMPK-PARP1 cascade, as shown in the current study. Therefore, AMPK phosphorylation of PARP1 would counteract other phosphorylation events activating PARP1.

Noticeably, statins (known to activate AMPK) [49] and other ARBs may also activate this beneficial pathway because atorvastatin and losartan increased PARP1 phosphorylation in ECs. On the other hand, other sulfonylurea drugs and β-blockers lack this beneficial effect (S6 and S7 Figs). While our study provides molecular basis of additional vascular beneficial effect of metformin and telmisartan in hyperglycemic or hypertensive patients, these results by no means challenge the clinical use of metoprolol and glipizide. Rather, given that metoprolol has profound effect in treating patients with heart failure, ischemic heart disease, or arrhythmia, combinatory use of drugs such as telmisartan or statins may provide additional vascular protection. Likewise, the combined use of glipizide, metformin, and statins may show pleiotropic effect in the vessels of patient with diabetes through activation of the AMPK-PARP1 axis.

## Supporting Information

S1 Fig High glucose decreased AMPK phosphorylation of PARP1 Ser-177 in HUVECs. (PDF)

S2 Fig Metformin or Telmisartan, but not Glipizide and Metoprolol, reduced PARP1 activity induced by high glucose or Ang II. (PDF)

S3 Fig Metformin or glipizide lowered blood glucose in db/db mice. (PDF)

S4 Fig Telmisartan or Metoprolol decreased blood pressure in SHR. (PDF)

S5 Fig Phenformin increased AMPK phosphorylation of PARP1 Ser-177 in HUVECs compared to glimepiride. (PDF)

S6 Fig Losartan enhanced AMPK phosphorylation of PARP1 Ser-177 in HUVECs compared to atenolol. (PDF)

S7 Fig Atorvastatin augmented AMPK phosphorylation of PARP1 Ser-177 in HUVECs. (PDF)

## References

[1] Jagtap P, Szabo C. Poly(ADP-ribose) polymerase and the therapeutic effects of its inhibitors. Nat Rev Drug Discov. 2005;4: 421–440. 15864271 10.1038/nrd1718

[2] Rouleau M, Patel A, Hendzel MJ, Kaufmann SH, Poirier GG. PARP inhibition: PARP1 and beyond. Nat Rev Cancer. 2010; 10: 293–301. 10.1038/nrc2812 20200537 PMC2910902

[3] Oliver FJ, Menissier-de Murcia J, Nacci C, Decker P, Andriantsitohaina R, Muller S, et al Resistance to endotoxic shock as a consequence of defective NF-kappaB activation in poly (ADP-ribose) polymerase-1 deficient mice. EMBO J. 1999;18: 4446–4454. 10449410 10.1093/emboj/18.16.4446 PMC1171519

[4] Andreone TL, O'Connor M, Denenberg A, Hake PW, Zingarelli B. Poly(ADP-ribose) polymerase-1 regulates activation of activator protein-1 in murine fibroblasts. J Immunol. 2003;170: 2113–2120. 12574383 10.4049/jimmunol.170.4.2113

[5] Bai P, Canto C, Oudart H, Brunyánszki A, Cen Y, Thomas C, et al PARP-1 inhibition increases mitochondrial metabolism through SIRT1 activation. Cell Metab. 2011;13: 461–468. 10.1016/j.cmet.2011.03.004 21459330 PMC3086520

[6] Ambrose HE, Papadopoulou V, Beswick RW, Wagner SD. Poly-(ADP-ribose) polymerase-1 (Parp-1) binds in a sequence-specific manner at the Bcl-6 locus and contributes to the regulation of Bcl-6 transcription. Oncogene. 2007;26: 6244–6252. 17404575 10.1038/sj.onc.1210434

[7] Pacher P, Szabo C. Role of the peroxynitrite-poly(ADP-ribose) polymerase pathway in human disease. Am J Pathol. 2008;173: 2–13. 10.2353/ajpath.2008.080019 18535182 PMC2438280

[8] Mathews MT, Berk BC. PARP-1 inhibition prevents oxidative and nitrosative stress-induced endothelial cell death via transactivation of the VEGF receptor 2. Arterioscler Thromb Vasc Biol. 2008; 28:711–717. 10.1161/ATVBAHA.107.156406 18239155

[9] Soriano FG, Pacher P, Mabley J, Liaudet L, Szabo C. Rapid reversal of the diabetic endothelial dysfunction by pharmacological inhibition of poly(ADP-ribose) polymerase. Circ Res. 2001;89: 684–691. 11597991 10.1161/hh2001.097797

[10] von Lukowicz T, Hassa PO, Lohmann C, Borén J, Braunersreuther V, Mach F, et al PARP1 is required for adhesion molecule expression in atherogenesis. Cardiovasc Res. 2008;78: 158–166. 18093987 10.1093/cvr/cvm110

[11] Oumouna-Benachour K, Hans CP, Suzuki Y, Naura A, Datta R, Belmadani S, et al Poly(ADP-ribose) polymerase inhibition reduces atherosclerotic plaque size and promotes factors of plaque stability in apolipoprotein E-deficient mice: effects on macrophage recruitment, nuclear factor-kappaB nuclear translocation, and foam cell death. Circulation. 2007; 115: 2442–2450. 17438151 10.1161/CIRCULATIONAHA.106.668756

[12] Hans CP, Feng Y, Naura AS, Zerfaoui M, Rezk BM, Xia H, et al Protective effects of PARP-1 knockout on dyslipidemia-induced autonomic and vascular dysfunction in ApoE mice: effects on eNOS and oxidative stress. PloS One. 2009;4: e7430 10.1371/journal.pone.0007430 19823587 PMC2757717

[13] Libby P. Metformin and vascular protection: a cardiologist's view. Diabetes Metab. 2003;29: 6S117–120. 14502109 10.1016/s1262-3636(03)72796-7

[14] de Aguiar LG, Bahia LR, Villela N, Laflor C, Sicuro F, Wiernsperger N, et al Metformin improves endothelial vascular reactivity in first-degree relatives of type 2 diabetic patients with metabolic syndrome and normal glucose tolerance. Diabetes Care. 2006;29: 1083–1089. 16644641 10.2337/diacare.2951083

[15] Benndorf RA, Appel D, Maas R, Schwedhelm E, Wenzel UO, Boger RH. Telmisartan improves endothelial function in patients with essential hypertension. J Cardiovasc Pharm. 2007;50: 367–371. 10.1097/FJC.0b013e31811dfbe7 18049303

[16] Asmar R, Gosse P, Topouchian J, N'Tela G, Dudley A, Shepherd GL. Effects of telmisartan on arterial stiffness in Type 2 diabetes patients with essential hypertension. Journal of the renin-angiotensin-aldosterone system: JRAAS. 2002;3: 176–180. 12563568 10.3317/jraas.2002.038

[17] Matsumura T, Kinoshita H, Ishii N, Fukuda K, Motoshima H, Senokuchi T, et al Telmisartan exerts antiatherosclerotic effects by activating peroxisome proliferator-activated receptor-gamma in macrophages. Arterioscler Thromb Vasc Biol. 2011;31: 1268–1275. 10.1161/ATVBAHA.110.222067 21474824

[18] Higashi Y, Sasaki S, Nakagawa K, Ueda T, Yoshimizu A, Kurisu S, et al A comparison of angiotensin-converting enzyme inhibitors, calcium antagonists, beta-blockers and diuretic agents on reactive hyperemia in patients with essential hypertension: a multicenter study. J Am Coll Cardiol. 2000;35: 284–291. 10676671 10.1016/s0735-1097(99)00561-6

[19] Fisslthaler B, Fleming I. Activation and signaling by the AMP-activated protein kinase in endothelial cells. Circ Res. 2009;105: 114–127. 10.1161/CIRCRESAHA.109.201590 19608989

[20] Gongol B, Marin T, Peng IC, Woo B, Martin M, King S, et al AMPKalpha2 exerts its anti-inflammatory effects through PARP-1 and Bcl-6. Proc Natl Acad Sci USA. 2013;110: 3161–3166. 10.1073/pnas.1222051110 23382195 PMC3581905

[21] Ido Y, Carling D, Ruderman N. Hyperglycemia-induced apoptosis in human umbilical vein endothelial cells: inhibition by the AMP-activated protein kinase activation. Diabetes. 2002;51: 159–167. 11756336 10.2337/diabetes.51.1.159

[22] Deji N, Kume S, Araki S, Isshiki K, Araki H, Chin-Kanasaki M, et al Role of angiotensin II-mediated AMPK inactivation on obesity-related salt-sensitive hypertension. Biochem Biophys Res Commun. 2012;418: 559–564. 10.1016/j.bbrc.2012.01.070 22293193

[23] Hong J, Zhang Y, Lai S, Lv A, Su Q, Dong Y, et al SPREAD-DIMCAD Investigators. Effects of metformin versus glipizide on cardiovascular outcomes in patients with type 2 diabetes and coronary artery disease. Diabetes Care. 2013;36: 1304–1311. 10.2337/dc12-0719 23230096 PMC3631843

[24] Pacher P, Liaudet L, Soriano FG, Mabley JG, Szabo E, Szabo C. The role of poly(ADP-ribose) polymerase activation in the development of myocardial and endothelial dysfunction in diabetes. Diabetes. 2002;51: 514–521. 11812763 10.2337/diabetes.51.2.514

[25] Choi SK, Galan M, Kassan M, Partyka M, Trebak M, Matrougui K. Poly(ADP-ribose) polymerase 1 inhibition improves coronary arteriole function in type 2 diabetes mellitus. Hypertension. 2012;59: 1060–1068. 10.1161/HYPERTENSIONAHA.111.190140 22454481 PMC3331962

[26] Effect of intensive blood-glucose control with metformin on complications in overweight patients with type 2 diabetes (UKPDS 34). UK Prospective Diabetes Study (UKPDS) Group. Lancet. 1998;352: 854–865. 9742977

[27] Holman RR, Paul SK, Bethel MA, Neil HA, Matthews DR. Long-term follow-up after tight control of blood pressure in type 2 diabetes. New Engl J Med. 2008;359: 1565–1576. 10.1056/NEJMoa0806359 18784091

[28] Mather KJ, Verma S, Anderson TJ. Improved endothelial function with metformin in type 2 diabetes mellitus. J Am Coll Cardiol. 2001;37: 1344–1350. 11300445 10.1016/s0735-1097(01)01129-9

[29] Boyle JG, Logan PJ, Jones GC, Small M, Sattar N, Connell JM, et al AMP-activated protein kinase is activated in adipose tissue of individuals with type 2 diabetes treated with metformin: a randomised glycaemia-controlled crossover study. Diabetologia. 2011;54: 1799–1809. 10.1007/s00125-011-2126-4 21455728

[30] Cheang WS, Tian XY, Wong WT, Lau CW, Lee SS, Chen ZY, et al Metformin protects endothelial function in diet-induced obese mice by inhibition of endoplasmic reticulum stress through 5’ adenosine monophosphate-activated protein kinase-peroxisome proliferator-activated receptor delta pathway. Arterioscler Thromb Vasc Biol. 2014;34: 830–836. 10.1161/ATVBAHA.113.301938 24482374

[31] Diamanti-Kandarakis E. Reproductive endocrinology: Infertility treatment in PCOS—is metformin in from the cold? Nat Rev Endocrinol. 2012;8: 328–330. 10.1038/nrendo.2012.69 22547260

[32] Paradisi G, Steinberg HO, Hempfling A, Cronin J, Hook G, Shepard MK, et al Polycystic ovary syndrome is associated with endothelial dysfunction. Circulation. 2001;103: 1410–1415. 11245645 10.1161/01.cir.103.10.1410

[33] Romualdi D, Costantini B, Selvaggi L, Giuliani M, Cristello F, Macrì F, et al Metformin improves endothelial function in normoinsulinemic PCOS patients: a new prospective. Hum Reprod. 2008;23: 2127–2133. 10.1093/humrep/den230 18567896

[34] James PA, Oparil S, Carter BL, Cushman WC, Dennison-Himmelfarb C, Handler J, et al 2014 evidence-based guideline for the management of high blood pressure in adults: report from the panel members appointed to the Eighth Joint National Committee (JNC 8). JAMA. 2014;311: 507–520. 10.1001/jama.2013.284427 24352797

[35] Bellien J, Iacob M, Eltchaninoff H, Bourkaib R, Thuillez C, Joannides R. AT1 receptor blockade prevents the decrease in conduit artery flow-mediated dilatation during NOS inhibition in humans. Clin Sci (Lond). 2007;112: 393–401. 17125466 10.1042/CS20060236

[36] Kishi T, Hirooka Y, Konno S, Sunagawa K. Angiotensin II receptor blockers improve endothelial dysfunction associated with sympathetic hyperactivity in metabolic syndrome. J Hypertens. 2012;30: 1646–1655. 10.1097/HJH.0b013e328355860e 22728908

[37] Schiffrin EL, Park JB, Pu Q. Effect of crossing over hypertensive patients from a beta-blocker to an angiotensin receptor antagonist on resistance artery structure and on endothelial function. J Hypertens. 2002;20: 71–78. 11791028 10.1097/00004872-200201000-00011

[38] Schiffrin EL, Park JB, Intengan HD, Touyz RM. Correction of arterial structure and endothelial dysfunction in human essential hypertension by the angiotensin receptor antagonist losartan. Circulation. 2000;101: 1653–1659. 10758046 10.1161/01.cir.101.14.1653

[39] Dorenkamp M, Riad A, Stiehl S, Spillmann F, Westermann D, Du J, et al Protection against oxidative stress in diabetic rats: role of angiotensin AT(1) receptor and beta 1-adrenoceptor antagonism. Eur J Pharmacol. 2005;520: 179–187. 16139267 10.1016/j.ejphar.2005.07.020

[40] Satoh M, Haruna Y, Fujimoto S, Sasaki T, Kashihara N. Telmisartan improves endothelial dysfunction and renal autoregulation in Dahl salt-sensitive rats. Hypertens Res. 2010;33: 135–142. 10.1038/hr.2009.190 19927153

[41] Scalera F, Martens-Lobenhoffer J, Bukowska A, Lendeckel U, Tager M, Bode-Boger SM. Effect of telmisartan on nitric oxide—asymmetrical dimethylarginine system: role of angiotensin II type 1 receptor gamma and peroxisome proliferator activated receptor gamma signaling during endothelial aging. Hypertension. 2008;51: 696–703. 10.1161/HYPERTENSIONAHA.107.104570 18250362

[42] Yuen CY, Wong WT, Tian XY, Wong SL, Lau CW, Yu J, et al Telmisartan inhibits vasoconstriction via PPARgamma-dependent expression and activation of endothelial nitric oxide synthase. Cardiovasc Res. 2011;90: 122–129. 10.1093/cvr/cvq392 21156825

[43] Sharma V, Dhillon P, Wambolt R, Parsons H, Brownsey R, Allard MF, et al Metoprolol improves cardiac function and modulates cardiac metabolism in the streptozotocin-diabetic rat. Am J Physiol-Heart Circ Physiol. 2008;294:H1609–1620. 10.1152/ajpheart.00949.2007 18203848

[44] Racz B, Hanto K, Tapodi A, Solti I, Kalman N, Jakus P, et al Regulation of MKP-1 expression and MAPK activation by PARP-1 in oxidative stress: a new mechanism for the cytoplasmic effect of PARP-1 activation. Free Radic Biol Med. 2010;49: 1978–1988. 10.1016/j.freeradbiomed.2010.09.026 20920579

[45] Cohen-Armon M, Visochek L, Rozensal D, Kalal A, Geistrikh I, Klein R, et al DNA-independent PARP-1 activation by phosphorylated ERK2 increases Elk1 activity: a link to histone acetylation. Mol Cell. 2007;25: 297–308. 17244536 10.1016/j.molcel.2006.12.012

[46] Kauppinen TM, Chan WY, Suh SW, Wiggins AK, Huang EJ, Swanson RA. Direct phosphorylation and regulation of poly(ADP-ribose) polymerase-1 by extracellular signal-regulated kinases 1/2. Proc Natl Acad Sci USA. 2006;103: 7136–7141. 16627622 10.1073/pnas.0508606103 PMC1459030

[47] Qin WD, Wei SJ, Wang XP, Wang J, Wang WK, Liu F, et al Poly(ADP-ribose) polymerase 1 inhibition protects against low shear stress induced inflammation. Biochim Biophys Acta. 2013;1833: 59–68. 10.1016/j.bbamcr.2012.10.013 23085506

[48] Zhang S, Lin Y, Kim YS, Hande MP, Liu ZG, Shen HM. c-Jun N-terminal kinase mediates hydrogen peroxide-induced cell death via sustained poly(ADP-ribose) polymerase-1 activation. Cell Death Differ. 2007; 14: 1001–1010. 17218956 10.1038/sj.cdd.4402088

[49] Sun W, Lee TS, Zhu M, Gu C, Wang Y, Zhu Y, et al Statins activate AMP-activated protein kinase in vitro and in vivo. Circulation. 2006;114: 2655–2662. 17116771 10.1161/CIRCULATIONAHA.106.630194

