# Supplementary material for: Cardiovascular Protective Effect of Metformin and Telmisartan: Reduction of PARP1 Activity via the AMPK-PARP1 Cascade
Source: PLoS One. 2016 Mar 17;11(3):e0151845. doi: 10.1371/journal.pone.0151845 (PMC4795690; doi:10.1371/journal.pone.0151845)
Supplement: S1 Fig — (PDF) [file pone.0151845.s001.pdf]

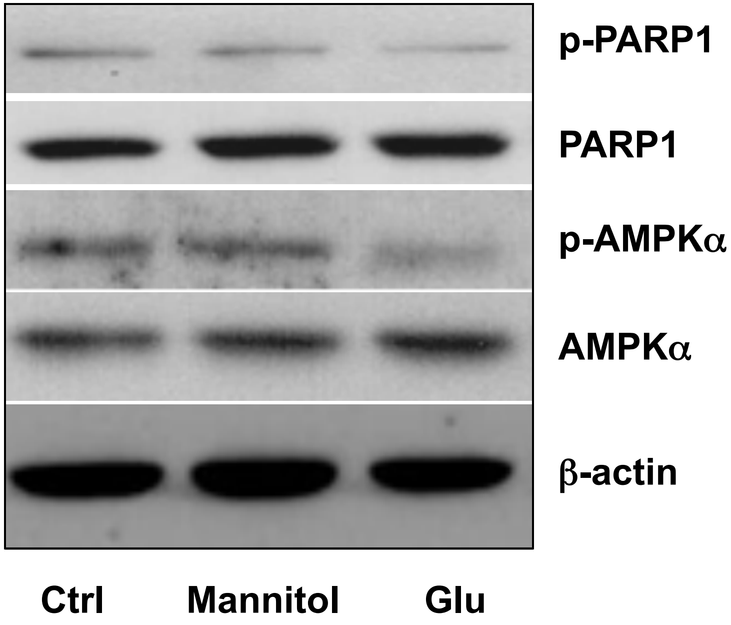

S1 Fig. High glucose decreased AMPK phosphorylation of PARP1 Ser-177 in HUVECs. Western blot analysis of AMPK Thr-172 and PARP1 Ser-177 in HUVECs treated with 30mM mannitol or 30 mM glucose for 4 hr.
