# Supplementary material for: Cardiovascular Protective Effect of Metformin and Telmisartan: Reduction of PARP1 Activity via the AMPK-PARP1 Cascade
Source: PLoS One. 2016 Mar 17;11(3):e0151845. doi: 10.1371/journal.pone.0151845 (PMC4795690; doi:10.1371/journal.pone.0151845)
Supplement: S2 Fig — (PDF) [file pone.0151845.s002.pdf]

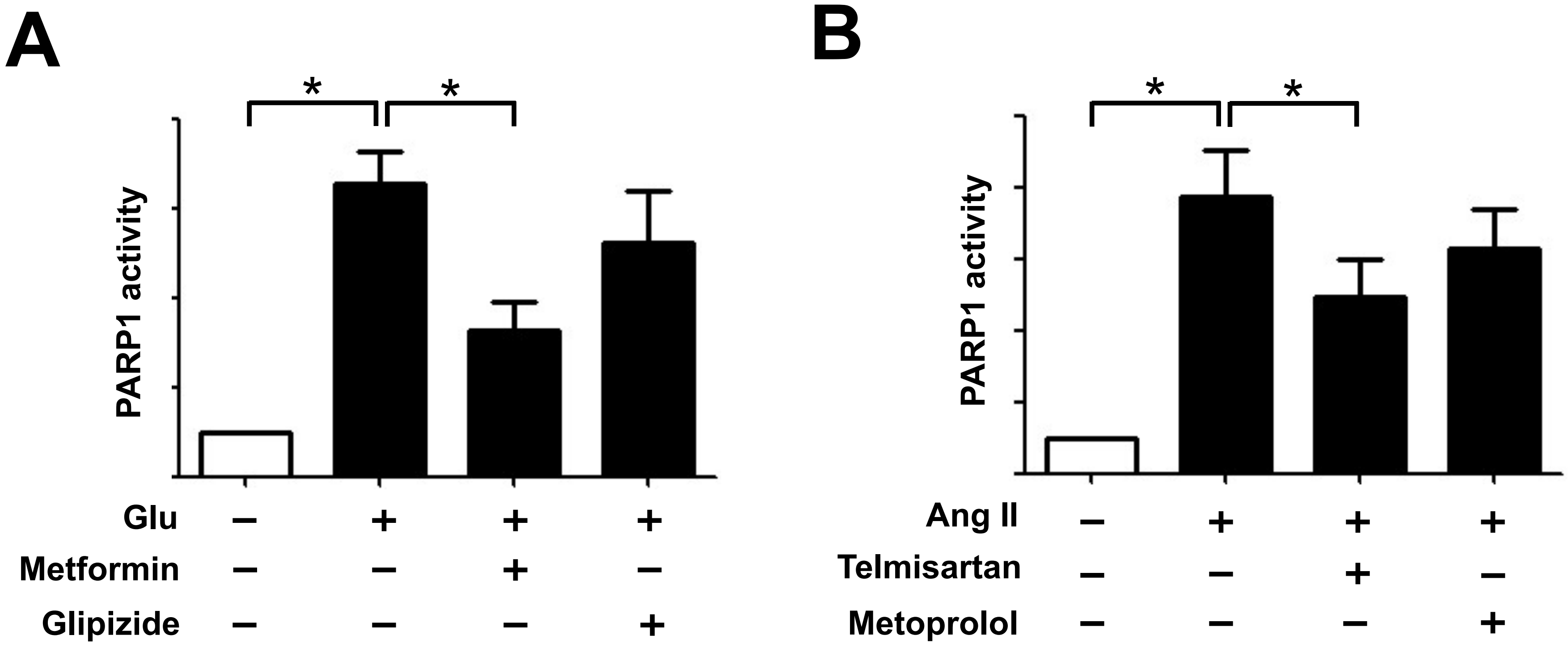

S2 Fig. Metformin or telmisartan, but not glipizide and metoprolol, reduced PARP1 activity induced by high glucose or Ang II. (A) HUVECs were treated with or without metformin or glipizide for 6 hr, then incubated with or without 30 mM glucose for 24 hr. (B) HUVECs were treated with or without telmisartan or metoprolol for 6 hr, then incubated with or without 100 nM Ang II for 24 hr (B). PARP1 activity in nuclear extracts of HUVECs was measured accordingly.
