# Supplementary material for: Cardiovascular Protective Effect of Metformin and Telmisartan: Reduction of PARP1 Activity via the AMPK-PARP1 Cascade
Source: PLoS One. 2016 Mar 17;11(3):e0151845. doi: 10.1371/journal.pone.0151845 (PMC4795690; doi:10.1371/journal.pone.0151845)
Supplement: S3 Fig — (PDF) [file pone.0151845.s003.pdf]

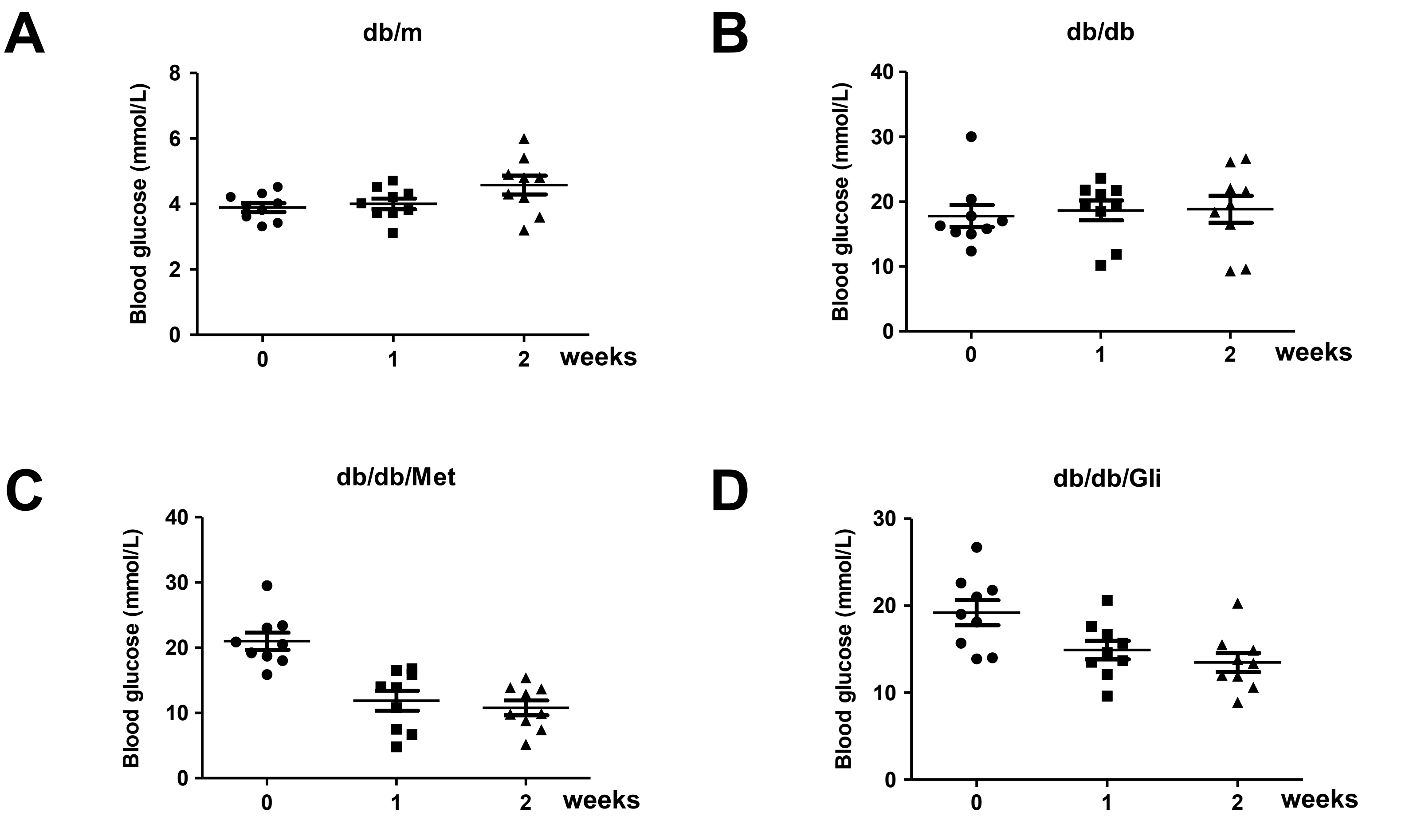

S3 Fig. Metformin or glipizide lowered blood glucose in db/db mice. Scatterplots of fasting blood glucose level in various mouse groups. (**A**) db/m mice and (**B,C,D**) db/db mice were treated with saline (A,B), metformin (200 mg/kg/day) (C) or glipizide (1.3mg/kg/day) (D) for 2 weeks. Fasting blood glucose level was measured at the beginning of the experiment (0 week), 1, and 2 weeks after drug administration.
