# Supplementary material for: Cardiovascular Protective Effect of Metformin and Telmisartan: Reduction of PARP1 Activity via the AMPK-PARP1 Cascade
Source: PLoS One. 2016 Mar 17;11(3):e0151845. doi: 10.1371/journal.pone.0151845 (PMC4795690; doi:10.1371/journal.pone.0151845)
Supplement: S4 Fig — (PDF) [file pone.0151845.s004.pdf]

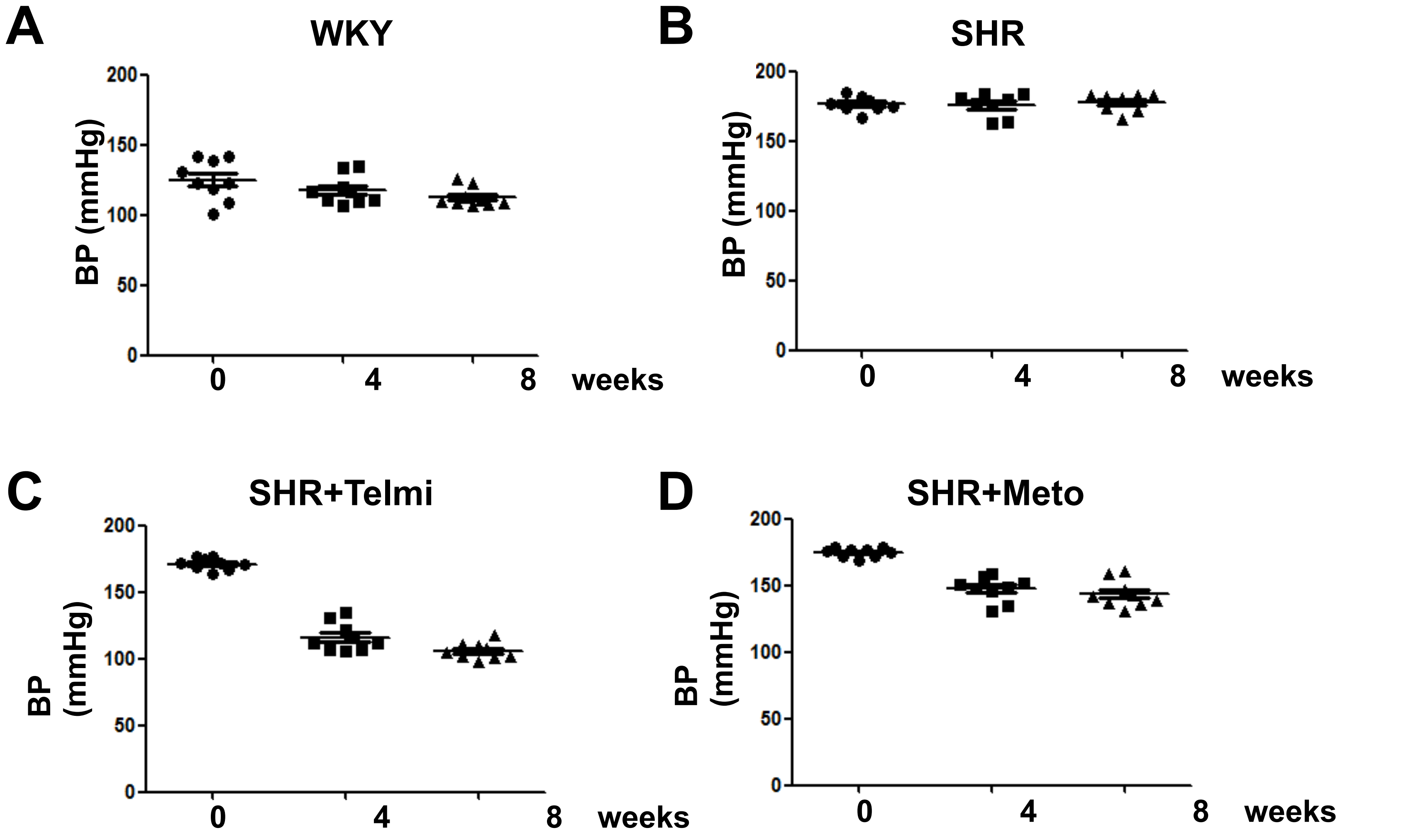

S4 Fig. Telmisartan and metoprolol decreased blood pressure in SHR. Scatterplots of blood pressure in WKY and SHR rats. (**A**) WKY and (**B,C,D**) SHR rats were treated with saline (A,B), telmisartan (10 mg/kg/day) (C) or metoprolol (30 mg/kg/day) (D) for 8 weeks. Systolic blood pressure level was measured at the beginning of the experiment (0 week), 4, and 8 weeks after drug administration.
