# Supplementary material for: Cardiovascular Protective Effect of Metformin and Telmisartan: Reduction of PARP1 Activity via the AMPK-PARP1 Cascade
Source: PLoS One. 2016 Mar 17;11(3):e0151845. doi: 10.1371/journal.pone.0151845 (PMC4795690; doi:10.1371/journal.pone.0151845)
Supplement: S5 Fig — (PDF) [file pone.0151845.s005.pdf]

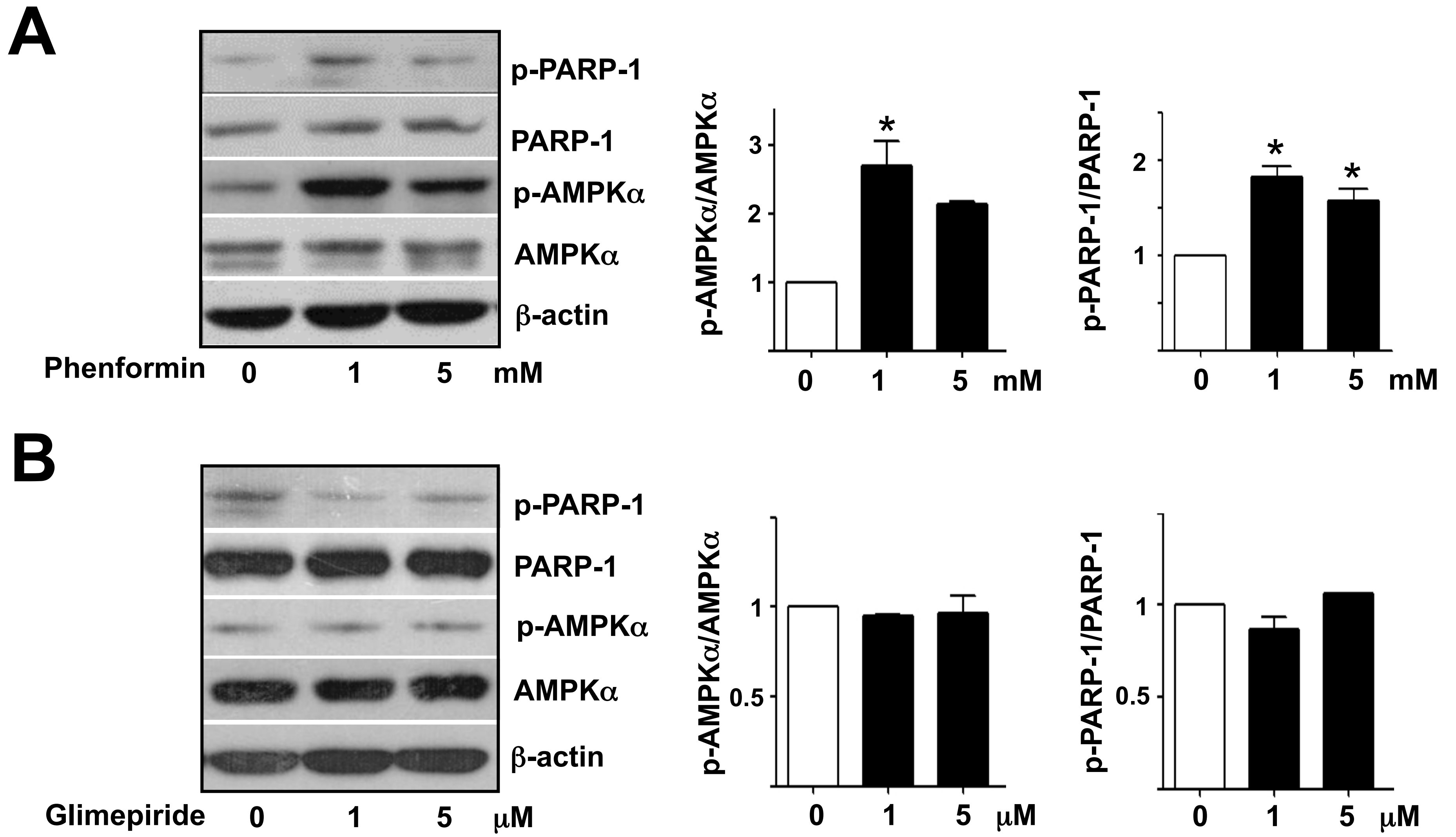

S5 Fig. Phenformin increased AMPK phosphorylation of PARP1 Ser-177 in HUVECs compared to glimepiride. Western blot analysis of AMPK Thr-172 and PARP1 Ser-177 phosphorylation in HUVECs treated with phenformin (**A**) or glimepiride (**B**) for 4 hr.
