# Supplementary material for: Cardiovascular Protective Effect of Metformin and Telmisartan: Reduction of PARP1 Activity via the AMPK-PARP1 Cascade
Source: PLoS One. 2016 Mar 17;11(3):e0151845. doi: 10.1371/journal.pone.0151845 (PMC4795690; doi:10.1371/journal.pone.0151845)
Supplement: S7 Fig — (PDF) [file pone.0151845.s007.pdf]

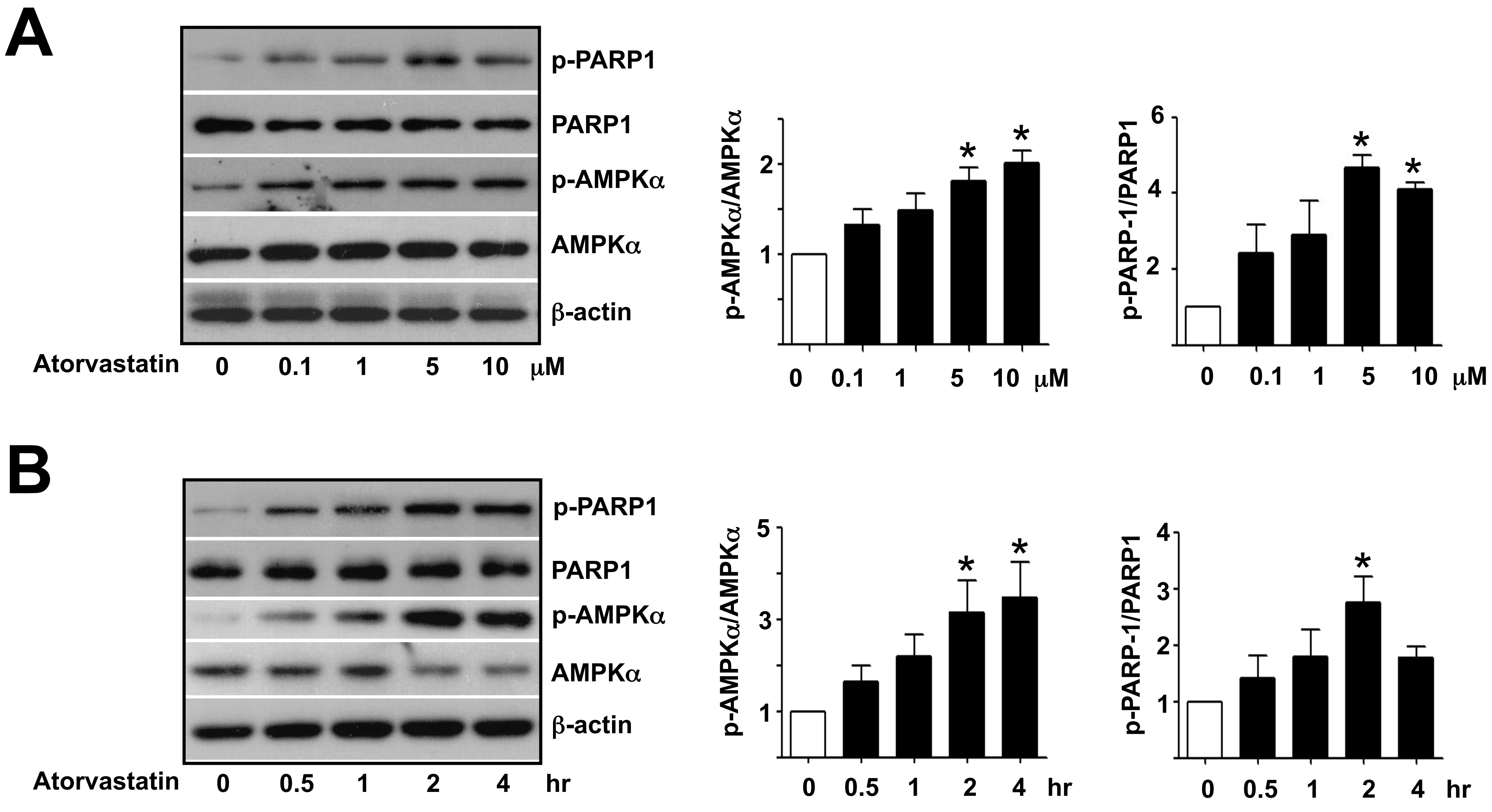

S7 Fig. Atorvastatin augmented AMPK phosphorylation of PARP1 Ser-177 in HUVECs. (**A**) HUVECs were treated with atorvastatin with indicated concentrations for 6 hr. (**B**) HUVECs were treated with atorvastatin (5 μM) for the time indicated. Western blotting was performed with various antibodies.
